# Catecholamine‐Secreting Retroperitoneal Paraganglioma: A Case Report and Literature Review

**DOI:** 10.1155/criu/2760597

**Published:** 2026-10-11

**Authors:** Eduardo Felício, Nuno Gião, Guilherme Bernardo, André Barcelos, Sara Duarte, Filipe Gaboleiro, André Pita, Diogo Carmali, Diogo Saraiva, Catarina Roque, João Varregoso, Fernando Ferrito

**Affiliations:** ^1^ Urology Department ULS Amadora-Sintra Amadora Portugal; ^2^ Pathology Department ULS São José Lisbon Portugal

**Keywords:** paraganglioma, retroperitoneal neoplasm, surgery

## Abstract

**Introduction:**

Paragangliomas are rare neuroendocrine tumors arising from autonomic paraganglia. These tumors have metastatic potential, and histopathological findings alone cannot reliably predict their biological behavior. Clinical manifestations range from the classic triad of headache, diaphoresis, and palpitations to incidental findings in asymptomatic patients.

**Case Presentation:**

A 75‐year‐old man was incidentally found to have a 5 cm retroperitoneal mass adjacent to the right renal hilum. Biochemical testing confirmed marked catecholamine hypersecretion, and 18F‐FDOPA PET/CT demonstrated isolated uptake within the lesion without additional disease. Preoperative preparation included progressive alpha‐adrenergic blockade with doxazosin for approximately 1 month, followed by atenolol for heart rate control. An open approach through a right subcostal hemichevron incision was selected because the tumor was located between the right renal artery and vein and lateral to the inferior vena cava. During dissection, an iatrogenic renal artery injury with intraoperative hemorrhage occurred, necessitating en bloc removal of the tumor, right kidney, and right adrenal gland. Histopathological examination confirmed a paraganglioma with a Ki‐67 proliferation index below 1%, focal nuclear atypia, no mitotic figures, no tumor necrosis, no vascular invasion, and microscopic margin involvement. Immunohistochemistry was positive for Chromogranin A, synaptophysin, GATA‐3, and S100. Germline testing using a 14‐gene pheochromocytoma–paraganglioma panel identified no reported pathogenic or likely pathogenic variants. At 6 months, contrast‐enhanced thoracoabdominopelvic computed tomography showed no evidence of local recurrence or metastatic disease, and plasma and urinary metanephrines had normalized, consistent with postoperative biochemical remission. The patient remains under joint endocrinology and urology follow‐up with lifelong annual clinical and biochemical surveillance.

**Discussion and Conclusion:**

This case highlights the diagnostic and surgical challenges of a functioning perihilar retroperitoneal paraganglioma. Careful endocrine preparation, vascular planning, multidisciplinary management, and lifelong surveillance are essential, particularly when the surgical margin is microscopically involved.

## 1. Introduction

Paragangliomas are rare neuroendocrine tumors arising from autonomic paraganglia. Sympathetic paragangliomas, particularly those arising in the abdomen and retroperitoneum, may secrete catecholamines, although their clinical presentation is variable. According to current World Health Organization terminology, all paragangliomas are considered to have metastatic potential because histopathological findings alone cannot reliably predict their biological behavior [1, 2].

Retroperitoneal paragangliomas located near the renal hilum present particular surgical challenges because of their close relationship with the renal vessels and other major retroperitoneal structures. We report an incidentally diagnosed functioning paraganglioma, emphasizing preoperative endocrine preparation, surgical decision‐making, management of an intraoperative vascular complication, pathological assessment, and surveillance following microscopic margin involvement.

## 2. Case Presentation

A 75‐year‐old man with a medical history of hypertension and benign prostatic hyperplasia was referred to the Urology Department after the incidental identification of a retroperitoneal mass on a chest computed tomography scan performed during the investigation of pneumonia. His medication included lercanidipine, telmisartan/hydrochlorothiazide, and tamsulosin. At presentation, blood pressure was 128/83 mmHg, and heart rate was 73 beats per minute. The patient denied headache, palpitations, diaphoresis, or other paroxysmal symptoms suggestive of catecholamine excess. There was no known family history of pheochromocytoma, paraganglioma, or an associated hereditary tumor syndrome.

Abdominal MRI demonstrated a well‐circumscribed 5 cm retroperitoneal lesion located between the right renal artery and renal vein and lateral to the inferior vena cava. The lesion showed peripheral enhancement, displacement of the renal vein, a central nonenhancing area suggestive of necrosis, diffusion restriction, and marked T2 hyperintensity (“lightbulb sign”), features highly suspicious for paraganglioma.

Although the patient denied classic paroxysmal symptoms such as palpitations, headache, or diaphoresis, biochemical evaluation demonstrated marked catecholamine hypersecretion. Plasma metanephrine was 1086 pg/mL (reference value < 90 pg/mL), and plasma normetanephrine was 1158 pg/mL (reference value < 190 pg/mL). Twenty‐four‐hour urinary total metanephrines were 4454 *μ*g/24 h (reference range 103–1144 *μ*g/24 h), including metanephrine of 1956 *μ*g/24 h (reference value < 320 *μ*g/24 h) and normetanephrine of 2267 *μ*g/24 h (reference value < 390 *μ*g/24 h). Urinary 3‐methoxytyramine was within the reference range at 231 *μ*g/24 h (reference range 103–434 *μ*g/24 h). Baseline estimated glomerular filtration rate was 79 mL/min/1.73 m^2^.

Functional imaging with 18F‐FDOPA PET/CT demonstrated focal uptake within the retroperitoneal lesion, with no evidence of multifocal or metastatic disease (Figure 1).

**Figure 1:**
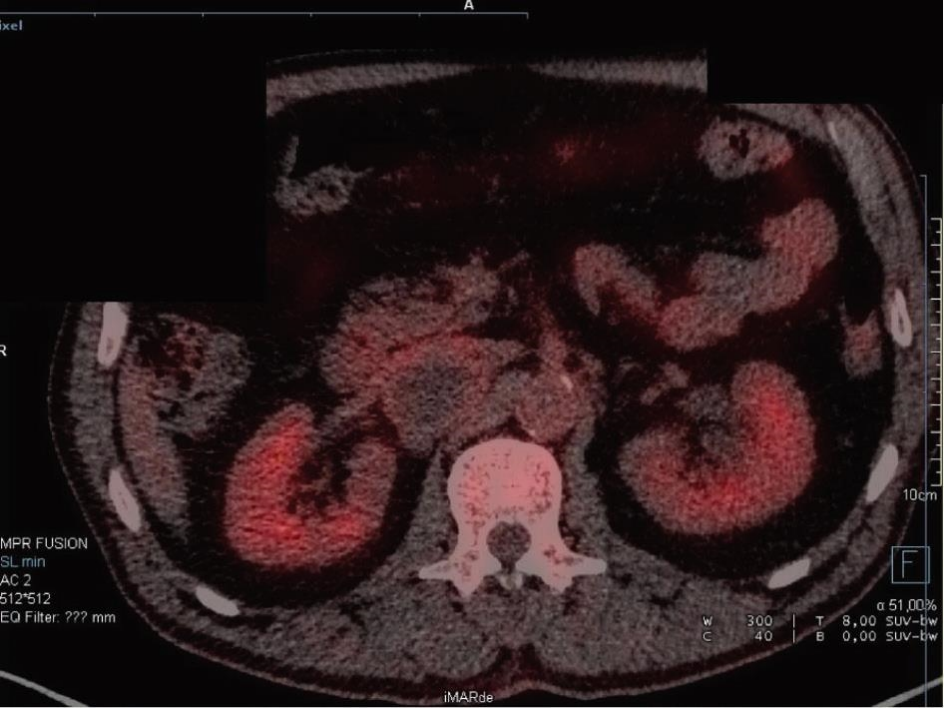
Axial 18F‐FDOPA PET/CT image showing focal radiotracer uptake in the right retroperitoneal lesion near the renal hilum, with no evidence of additional disease.

Preoperative medical preparation was performed for approximately 1 month. Doxazosin was progressively titrated from 4 mg twice daily to 6 mg twice daily, followed by 6 mg in the morning and 8 mg in the evening. Preparation continued for approximately 1 month to allow gradual outpatient dose adjustment according to blood pressure, heart rate, and tolerability. Atenolol 25 mg daily was introduced after alpha‐adrenergic blockade because of persistent resting sinus tachycardia, with heart rate measurements of 102–108 bpm. Seated blood pressure was 126/78 mmHg, and heart rate was 66 bpm. After 3 min of standing, blood pressure was 114/72 mmHg, and heart rate was 76 bpm. No orthostatic hypotension, dizziness, or presyncope occurred during preparation. Oral hydration was encouraged, dietary salt restriction was not prescribed, and no formal salt‐loading or intravenous volume‐expansion protocol was used.

An open surgical approach was selected because of the tumor′s perihilar location, allowing adequate exposure and vascular control. Surgery was performed through a right subcostal hemichevron incision (Figure 2). During tumor manipulation, mild hemodynamic fluctuations occurred and were adequately managed by the anesthesiology team, without clinically significant hypertensive episodes.

**Figure 2:**
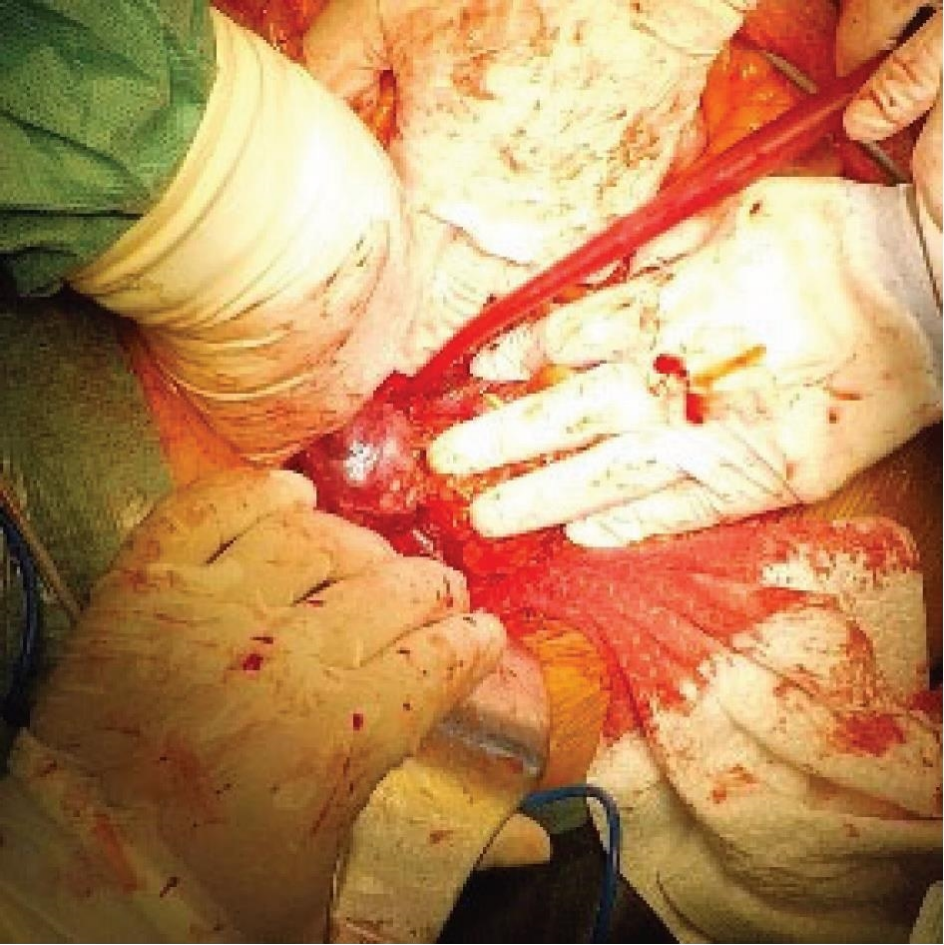
Intraoperative image showing isolation of the retroperitoneal paraganglioma before resection.

During dissection of the retroperitoneal paraganglioma, an iatrogenic injury to the right renal artery occurred, resulting in significant intraoperative hemorrhage. A vascular surgery team was present intraoperatively. Renal artery reconstruction by anastomosis was considered; however, the proximal renal artery stump was deemed too short to permit the proposed anastomosis. Right nephrectomy was therefore performed. In this context, due to hemorrhage, preservation of the right adrenal gland was not feasible, and the tumor, right kidney, and right adrenal gland were removed en bloc. The operative duration was approximately 3 h, with an estimated blood loss of 1000 mL. No blood transfusions were administered during surgery or postoperatively.

The postoperative course was generally favorable, and the patient was discharged after a 5‐day hospital stay. Approximately 1 month after surgery, orthostatic hypotension was documented. The patient also reported localized abdominal wall hypoesthesia, which progressively improved, with only mild residual sensory symptoms during follow‐up.

Histopathological examination demonstrated a neoplasm composed of epithelioid cells with granular cytoplasm arranged in variably sized nests, consistent with a zellballen growth pattern (Figure 3). Some nests were confluent, with focal areas of diffuse growth (Figure 4). Immunohistochemical staining was negative for Cytokeratin AE1/AE3 and CAM 5.2 and positive for Chromogranin A (Figure 5), synaptophysin, GATA‐3 (Figure 6), and S100. The Ki‐67 proliferation index was below 1%. Focal nuclear atypia was present, but no mitotic figures, tumor necrosis, or vascular invasion were identified. Microscopic tumor involvement of the surgical margin was present. The pathology report did not specify the anatomical location of margin involvement.

**Figure 3:**
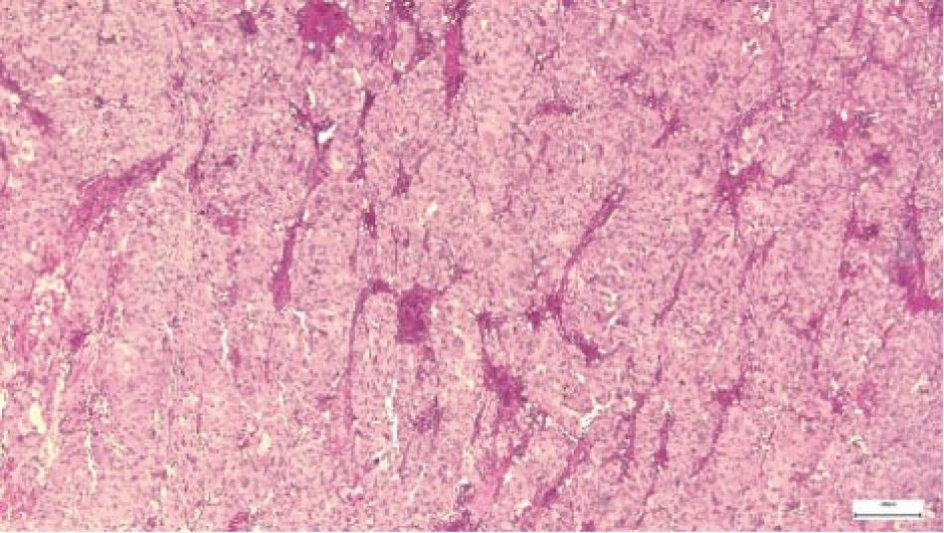
Neoplasm composed of epithelioid cells arranged in nests within a vascularized fibrous stroma, demonstrating a zellballen pattern (hematoxylin and eosin, ×50).

**Figure 4:**
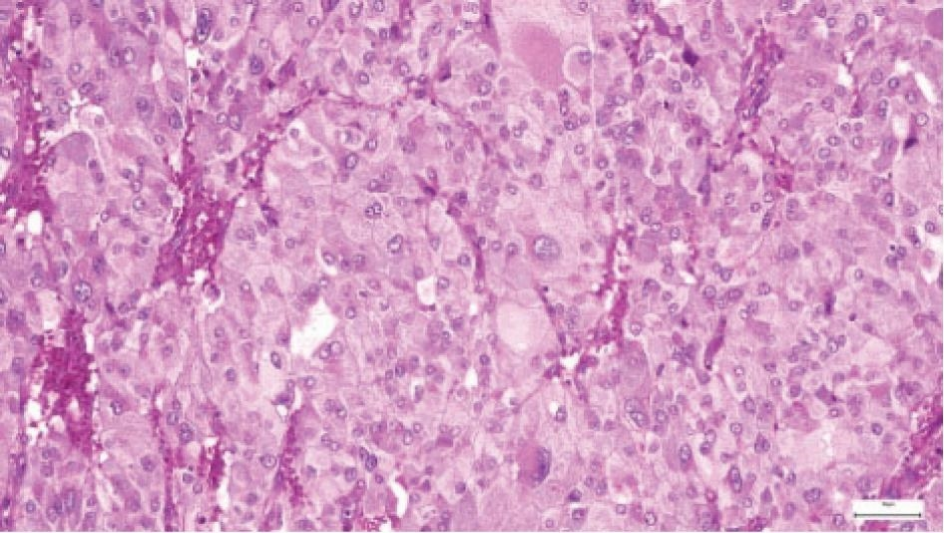
Tumor cells with finely granular eosinophilic cytoplasm and centrally located nuclei, with focal nuclear pleomorphism and occasional nucleoli (hematoxylin and eosin, ×200).

**Figure 5:**
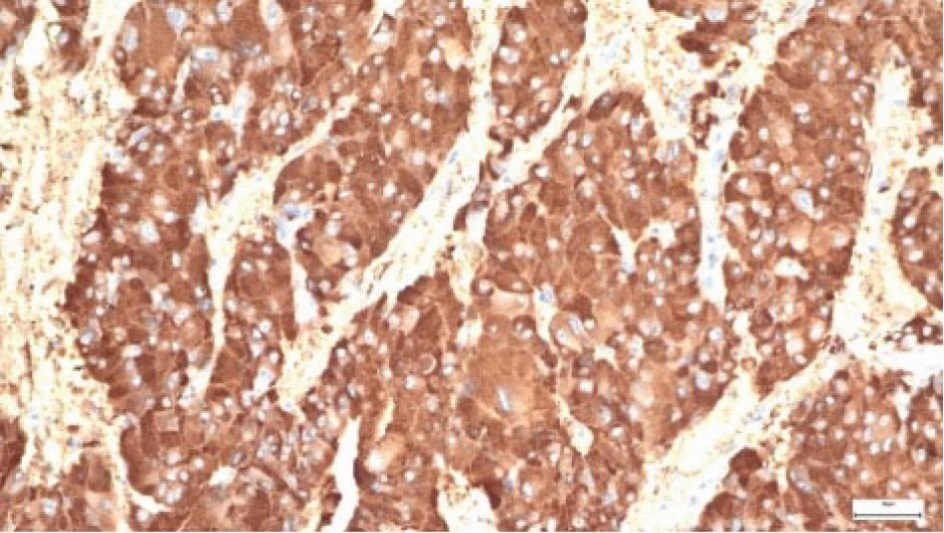
Diffuse cytoplasmic staining of neoplastic cells with Chromogranin A (×200).

**Figure 6:**
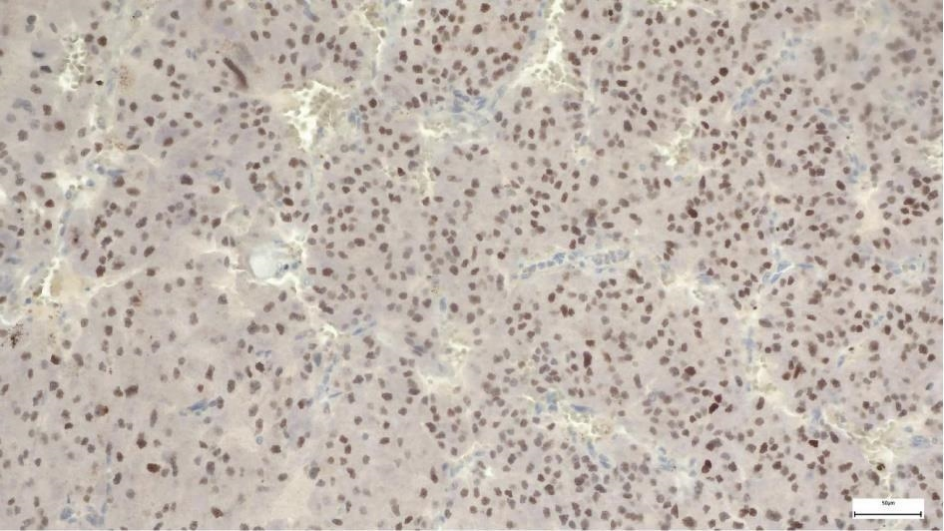
Nuclear immunoreactivity for GATA‐3 (×200).

The resection was considered macroscopically complete, with microscopic margin involvement consistent with the R1 resection. Given the absence of radiologically demonstrable residual or metastatic disease, no immediate additional treatment was undertaken, and clinical, biochemical, and radiological surveillance was selected.

Germline genetic testing was performed on EDTA‐anticoagulated blood using a 14‐gene pheochromocytoma–paraganglioma panel comprising FH, GDNF, KIF1B, MAX, MEN1, NF1, RET, SDHA, SDHAF2, SDHB, SDHC, SDHD, TMEM127, and VHL. Next‐generation sequencing was performed on an Illumina MiSeq platform, with alignment to the GRCh37/hg19 reference genome and bioinformatic analysis of copy‐number variants. No pathogenic or likely pathogenic variants, variants of uncertain significance, or copy‐number variants were reported in the analyzed genes.

Six months after surgery, contrast‐enhanced thoracoabdominopelvic computed tomography showed no evidence of local recurrence or metastatic disease. Plasma and urinary metanephrines had normalized, consistent with postoperative biochemical remission. Serum creatinine was 1.79 mg/dL at 6 months. A postoperative eGFR of 38.42 mL/min/1.73 m^2^ was also documented. The patient remains under joint endocrinology and urology follow‐up, with lifelong annual clinical and biochemical assessment. Annual thoracoabdominopelvic computed tomography surveillance is planned, with the next examination 12 months after the previous scan. The contrast protocol for subsequent annual CT examinations was not specified in the available follow‐up information.

## 3. Discussion

Paragangliomas are rare neuroendocrine tumors arising from autonomic paraganglia. Abdominal paragangliomas originate from the sympathetic paraganglia and may produce catecholamines, although their clinical presentation is highly variable. Patients may present with headache, diaphoresis, palpitations, and sustained or paroxysmal hypertension. However, functioning paragangliomas may also be diagnosed incidentally in patients without typical symptoms, as occurred in the present case despite markedly elevated plasma and urinary metanephrines [1, 3].

The diagnosis of sympathetic paraganglioma requires biochemical demonstration of catecholamine excess followed by anatomical and functional localization. In the present patient, magnetic resonance imaging identified a retroperitoneal mass in a particularly challenging perihilar location, positioned between the right renal artery and renal vein and lateral to the inferior vena cava. This close relationship with major vascular structures was an important consideration in operative planning and favored an open approach to provide adequate exposure and vascular control.

Functional imaging with 18F‐FDOPA PET/CT was performed to characterize the lesion and assess for multifocal or metastatic disease. 18F‐FDOPA is taken up by neuroendocrine cells through amino acid transport mechanisms and enters the catecholamine synthesis pathway. It is an established functional imaging option for pheochromocytomas and paragangliomas, and the choice of radiotracer should be individualized according to tumor location, biochemical phenotype, genotype, clinical purpose, and local availability [4]. In this case, 18F‐FDOPA PET/CT demonstrated isolated uptake within the retroperitoneal lesion, supporting surgical treatment with curative intent (Figure 1).

Surgical resection remains the cornerstone of treatment for localized and resectable paragangliomas [3]. Nevertheless, retroperitoneal paragangliomas may be highly vascular and intimately associated with the aorta, inferior vena cava, renal vessels, and other major retroperitoneal structures. Tumors involving the renal hilum are particularly demanding because dissection is performed in close proximity to both the renal artery and vein, with a risk of significant hemorrhage and loss of the renal unit [5]. In the present case, despite the open approach, renal artery injury occurred during dissection, resulting in intraoperative hemorrhage. A vascular surgery team was present, but the proximal renal artery stump was deemed too short to permit the proposed anastomosis, and nephrectomy was performed. The right adrenal gland could not be preserved in this setting, and the tumor, kidney, and adrenal gland were removed en bloc.

Preoperative medical preparation is essential in functioning paragangliomas because tumor manipulation may cause substantial catecholamine release and hemodynamic instability. Guidelines generally recommend 7–14 days of preoperative treatment, with titration according to blood pressure, heart rate, and tolerability [3]. In this patient, progressive doxazosin titration continued over approximately 1 month, followed by atenolol for heart rate control. Only mild intraoperative hemodynamic fluctuations occurred and were successfully managed by the anesthesiology team, without clinically significant hypertensive crises. This emphasizes the importance of multidisciplinary preparation involving endocrinology, anesthesiology, radiology, nuclear medicine, and the surgical team [3, 6].

Histopathological examination confirmed the diagnosis through the characteristic zellballen growth pattern and neuroendocrine immunophenotype (Figure 3), with positivity for Chromogranin A, synaptophysin, GATA‐3, and S100 and absence of epithelial cytokeratin expression. The Ki‐67 proliferation index was below 1%, and no mitotic figures, tumor necrosis, or vascular invasion were identified. Although these features indicate low proliferative activity, they cannot reliably exclude future recurrence or metastatic behavior. According to current World Health Organization terminology, all paragangliomas are considered to have metastatic potential, and the distinction between benign and malignant tumors should therefore be avoided [2].

Microscopic involvement of the surgical margin is clinically relevant because it may indicate residual microscopic disease. However, evidence regarding the independent prognostic significance of margin status in retroperitoneal paraganglioma remains limited. In a small retrospective series, survival was primarily associated with the development of metastatic disease rather than surgical margin status, although the sample size and retrospective design preclude definitive conclusions regarding local recurrence risk [7]. In the present case, surveillance was selected after macroscopically complete resection because no radiologically demonstrable residual or metastatic disease was identified. Subsequent normalization of plasma and urinary metanephrines and negative 6‐month imaging supported continued surveillance. These findings do not exclude microscopic residual disease. The involved margin could not be anatomically localized from the pathology report, which limits assessment of a potential target for further local treatment. Despite these favorable early results, microscopic margin involvement reinforces the need for particularly attentive, serial, and lifelong surveillance. The patient remains under joint endocrinology and urology follow‐up, with annual clinical and biochemical assessment and annual CT surveillance. The 6‐month CT was performed with intravenous contrast, and the next thoracoabdominopelvic CT examination is planned 12 months after that scan. MRI may be considered where appropriate to reduce cumulative radiation exposure [3]; the need for MRI contrast should also be assessed individually. This strategy is supported by the possibility of late recurrence or new tumor events after apparently successful surgery and by recommendations for lifelong annual follow‐up in patients with paragangliomas [8, 9].

## 4. Conclusion

This case highlights the diagnostic and therapeutic challenges of a functioning perihilar retroperitoneal paraganglioma. It emphasizes the importance of biochemical assessment in apparently asymptomatic patients, appropriate endocrine preparation, careful vascular planning, transparent reporting of operative complications, and lifelong multidisciplinary surveillance, particularly after microscopic margin involvement.

**Nomenclature**
18F‐FDOPA PET/CT: fluorine‐18‐labeled fluoro‐L‐DOPA positron emission tomography/computed tomography
CT: computed tomography
eGFR: estimated glomerular filtration rate
H&E: hematoxylin and eosin
MAX: MYC‐associated factor X
NF1: Neurofibromin 1
PGLs: paragangliomas
RET: rearranged during transfection
SDHx: succinate dehydrogenase subunits
VHL: Von Hippel–Lindau

## Author Contributions

**Eduardo Felício**: conception of the work, data collection, literature review, and drafting of the manuscript; **Guilherme Bernardo**, **André Barcelos**, **Sara Duarte**, **Filipe Gaboleiro**, **André Pita**, **Diogo Carmali**, **Diogo Saraiva**, **Catarina Roque**, **João Varregoso**, and **Fernando Ferrito**: supervision, critical revision of the manuscript, and overall oversight; **Nuno Gião**: histopathological analysis and contribution to manuscript revision.

## Funding

No funding was received for this manuscript.

## Disclosure

All authors have read and approved the final version of the manuscript. **Eduardo Felício** had full access to the clinical data and takes responsibility for the integrity and accuracy of the information presented.

## Ethics Statement

Formal ethics committee approval was not required for this single‐patient case report in accordance with institutional policy.

## Consent

Written informed consent was obtained from the patient for publication of this case report and all accompanying images.

## Conflicts of Interest

The authors declare no conflicts of interest.

## Data Availability

The data that support the findings of this study are available on request from the corresponding author. The data are not publicly available due to privacy or ethical restrictions.
